# The inert meets the living: The expanding view of metabolic alterations during viral pathogenesis

**DOI:** 10.1371/journal.ppat.1007830

**Published:** 2019-07-25

**Authors:** Karla D. Passalacqua, John G. Purdy, Christiane E. Wobus

**Affiliations:** 1 Department of Microbiology and Immunology, University of Michigan, Ann Arbor, Michigan, United States of America; 2 Department of Immunobiology, University of Arizona, Tucson, Arizona, United States of America; 3 BIO5 Institute, University of Arizona, Tucson, Arizona, United States of America; University of Wisconsin Madison, UNITED STATES

The term “metabolism” describes the thermodynamic underpinnings of living systems that have been investigated by scientists for centuries [1]. The study of metabolism in the “-omics age” has generated insights into the crucial metabolic elements coordinating complex cellular activities like immune responses, maintenance of the tumor microenvironment, autophagy, and the unique relationship between cells and viruses. Viruses possess the information-storage characteristic of life but lack the ability to extract free energy from nutrients. Therefore, viruses require host cell metabolism to perform the work needed for making new viral particles. As such, this particular host-pathogen interface is “unique” from a metabolic perspective, as a metabolically inert particle integrates into the metabolic landscape of a living cell to replicate noncellular particles.

Because viruses construct viral progeny from stolen biomolecules (e.g., amino acids, nucleotides, and sometimes lipids), the primary tier of research into the virus-host-metabolism relationship has focused on metabolic pathways that provide these needed materials. Two excellent reviews that highlight this tier of the virus-metabolism interface were published a few years ago [2, 3]. But an emerging tier of investigation seeks to uncover how metabolism affects the overall process of pathogenesis (Fig 1), expanding our view on the complexity of the virus-host metabolism interaction. In this Pearl, we will highlight four unique perspectives about the consequences of host metabolism on viral pathogenesis and disease progression.

**Fig 1:**
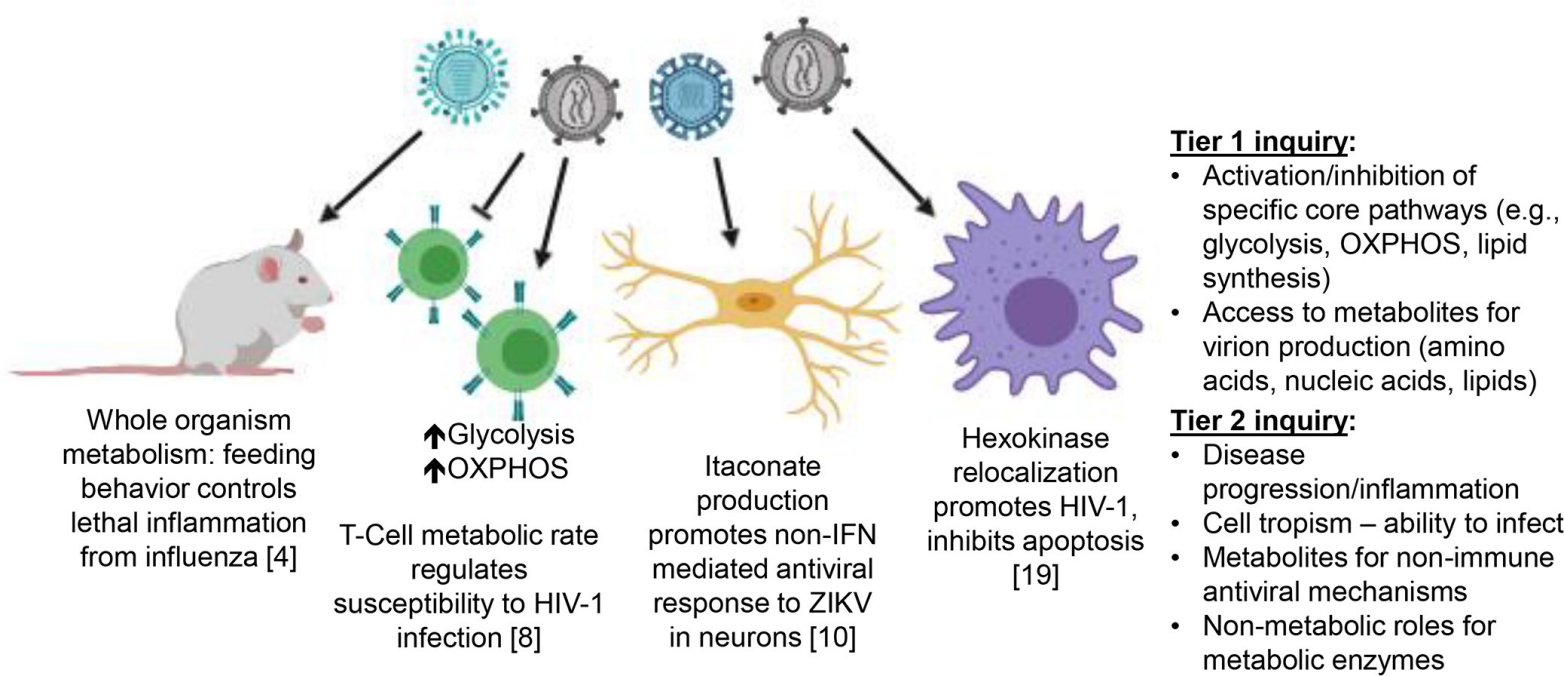
The expanding view of the role of cellular metabolism during viral infection. The initial track of inquiry into the metabolic manipulation of cells by viruses focused on identifying the core metabolic pathways that change during infection, and how these changes affect the availability of biomolecules for virus assembly (Tier 1 inquiry). New research is revealing how viral rewiring of host cell metabolism has even broader effects on the process of pathogenesis, including levels of inflammation, cell susceptibility to infection, viral clearance, and establishing viral reservoirs (Tier 2 inquiry). Viruses investigated were the influenza virus (light blue—[4]), HIV-1 (grey—[8, 19]), and Zika virus (dark blue—[10]). IFN, interferon; OXPHOS, oxidative phosphorylation; ZIKV, Zika virus.

## Eating versus fasting: How whole organism metabolism affects infectious disease progression

An ambitious study recently explored whether the old folk wisdom of “starving a fever and feeding a cold” has any biological validity [4]. In this work, Wang and colleagues juxtaposed viral infection with bacterial infection in a mouse model during a regimen of fasting versus feeding. This extensive study aimed to define the consequences that different whole-animal metabolic backgrounds have on inflammation caused by infection with the influenza virus, the bacterium *Listeria monocytogenes*, and also analogous proxies to these infections, poly(I:C) (double-stranded nucleic acid, a product of viral infection) and lipopolysaccharide (LPS; an inflammatory bacterial product). Overall, the study demonstrated opposing outcomes of fasting and feeding during the two infections. Specifically, higher glucose metabolism (feeding) was protective during viral infection but lethal during bacterial inflammation, whereas ketogenic metabolism (fasting) was protective during bacterial infection but detrimental during viral inflammation. The proposed model for this phenomenon suggests that viral inflammation and bacterial inflammation are qualitatively unique in terms of the glucose utilization patterns they induce and the ensuing inflammatory response. However, it remains unknown whether this simple model will hold for all viral and bacterial infections, because every infectious agent (and infectious proxies such as LPS and Poly(I:C)) can induce unique cellular/organismal responses. In this context, it is important to remember that studies about the effects of bacterial infection on mammalian cell metabolism may be confounded by the fact that bacteria are cellular and, unlike viruses, encode a metabolic network, and are confined by their own metabolic constraints. Therefore, it may be hard to disentangle just whose metabolism is being altered under experimental conditions, making the study of metabolism during viral infection easier to interpret. Indeed, immune activation with LPS has recently been shown to induce an energy preservation program in mice, where thermoregulation in turn modulates metabolic activity, generating a tolerance to inflammation [5]. This energy-saving program was shown to be protective during bacterial infection, but it is entirely unknown whether this immune system–induced protective metabolic program is also engaged during different viral infections.

These important studies provide a foundation for further exploration into the effects of feeding and fasting metabolism and the effect of immune-controlled thermoregulation on metabolism during other important viral and bacterial infections, which could have a powerful impact on the clinical treatment of various infectious diseases.

## A metabolic Achilles heel: HIV-1 preferentially infects cells exhibiting a specific metabolic state

Viruses are obligate intracellular pathogens that are highly adapted to productively infect certain cell types (i.e., viral tropism). Therefore, uncovering the host factors necessary for cell susceptibility is crucial for developing strategies to prevent viral infection. Investigating viral tropism for viruses that infect cells of the immune system is particularly challenging, because immune cells are highly plastic and able to differentiate temporally into a range of resting and active morphologies. Additionally, immune cells are further plastic in regard to their metabolic activity, as they adjust their metabolism to facilitate differentiation and effector function. Indeed, this phenomenon is the focus of a whole field of immunology dubbed “immunometabolism” [6, 7]. Recent work showed that immune cell metabolic plasticity is a key determinant in establishing susceptibility of T cells to infection by HIV-1 [8]. The authors of this study demonstrated that cluster of differentiation (CD)4^+^ T cells, which adopt a range of cellular states from naïve to effector T cells, are differentially susceptible to HIV-1 infection based on their levels of metabolic activity. Specifically, CD4^+^ cells exhibit a hierarchy of metabolic profiles of differing activity levels of glycolysis and oxidative phosphorylation (OXPHOS). This metabolic spectrum matches the cells’ susceptibility to HIV-1 infection, in which cells undergoing the highest levels of glycolysis and OXPHOS are the most susceptible, and those with the lowest are the least susceptible. This is a significant finding that not only defined the connection between levels of bioenergetic activity and susceptibility to viral infection for the first time but also demonstrated that inhibiting glucose metabolism blocks HIV-1 replication in infected cells and promotes the elimination of virus by enhancing cell death of infected cells. Thus, the traditional paradigm of viewing host cell susceptibility to viral infection in terms of the availability of specific protein/carbohydrate receptors can now be expanded to also include the underlying metabolic behavior of target cells as another host determinant of virus tropism. Future studies are needed to examine the variable susceptibility of other metabolically plastic cells to viruses and to determine whether this metabolic Achilles heel might be manipulated pharmacologically or through diet to prevent or eliminate infection.

## Antiviral metabolite production: Itaconate and the antiviral response

In addition to generating a cellular landscape that promotes viral infection, cellular metabolism can also be modulated to create an antiviral state. There is much evidence connecting cellular metabolism and the antiviral interferon response [9]. However, a recent study has uncovered a new antiviral metabolic program in neurons that limits Zika virus (ZIKV) infection that is independent of the canonical interferon response [10]. Flaviviruses like ZIKV, dengue virus, and West Nile virus cause severe disease if they gain access to the central nervous system (CNS). Through an in-depth investigation into the role of the protein receptor-interacting serine/threonine-protein kinase 3 (RIPK3) in limiting ZIKV replication in neurons, Daniels and colleagues uncovered that RIPK3 signaling in ZIKV-infected neurons leads to altered metabolism and increased levels of the molecule itaconate. Itaconate is a small molecule metabolite made by diverting aconitate away from the tricarboxylic acid (TCA cycle). Its role in modulating the inflammatory antibacterial response in macrophages has been well characterized [11–13], and it has also been shown to have an interferon (IFN)-induced antiviral role in macrophages infected with mimiviruses [14]. Also, an itaconate derivative was shown to have antiviral activity against the influenza virus [15]. For ZIKV-infected neurons, Daniels and colleagues showed that RIPK3 signaling did not result in cell death via necroptosis, as it has been shown to do previously [16, 17]. Rather, the signaling led to increased itaconate levels through increased expression of the enzyme immunoresponsive gene 1 (IRG1), also called aconitate decarboxylase 1. Itaconate restricted ZIKV infection in Ripk3^−/−^ and Irg1^−/−^ neurons, which are more susceptible to viral infection than wild-type cells. Importantly, the authors did not see changes in the expression of interferon-stimulated genes in the sensitive Ripk3^−/−^ ZIKV-infected cells or in mice, demonstrating that the antiviral response caused by itaconate was not due to an interferon response. Even more strikingly, exogenous itaconate injected intracranially in ZIKV-infected mice reduced brain viral burden, which has important implications for potential treatment of CNS infection. This study suggests that small molecule metabolites may have been repurposed during evolution in different cell types, depending on the unique vulnerabilities and roles of the cells. This is particularly significant for viruses like ZIKV, which can infect a range of cell types in different species, and can cause differential metabolic rewiring in human versus mosquito cells [18]. However, the full extent of the roles of itaconate in metabolism and antiviral mechanisms remains to be uncovered.

## Repurposing metabolic machinery to prevent cell death and establish a reservoir

The traditional way of envisioning metabolism is as a team of enzymes working in a linear pathway, in which each enzyme modifies a substrate and passes it on down the line for some specific metabolic goal. However, there is a growing appreciation for nonmetabolic roles of metabolic enzymes during infection, which can have major consequences to the life or death of the cell and to the immune response. It has been known that hexokinase (HK), the first enzyme in glycolysis, is predominantly physically associated with the outer surface of the mitochondrion. During HIV-1 infection of macrophages, this mitochondrial association is enhanced, and viral infection was observed to cause increased levels of mitochondria-bound HK [19]. However, despite higher protein levels, there was a concomitant decrease in HK enzymatic activity and a decrease in cell death by apoptosis. By inhibiting cell death by modifying localization and enzymatic activity of HK, the virus is able to use the macrophage as a reservoir, which is one of the reasons HIV infection is so difficult to treat. Conversely, relocalization of HK to the cytoplasm was shown to be important during bacterial infection, in which HK can physically detect the bacterial cell wall molecule N-acetylglucosamine [20] and trigger the NLR family pyrin domain containing 3 (NLRP3) inflammasome independent of enzymatic activity. There is an enormous variety of metabolic pathway enzyme profiles and activities in different cell types, and it is likely that there are other nonenzymatic roles that these proteins play during viral infection. Investigating these in the future promises to uncover new aspects of well-studied cellular players.

## Conclusions

It is now well established that viruses can drastically alter the metabolic behavior of the cells they infect, often promoting a cellular milieu that supports viral replication, and sometimes activating an antiviral state. When considering the larger process of viral pathogenesis, it is important to consider that all organisms, including those commonly used in research, have evolved specific metabolic programs that are tissue and species specific [21] and that there are many physiological states, such as diabetes and obesity, that alter an organism’s metabolic program, which in turn can affect susceptibility to viral infection (e.g., [22]). These avenues of inquiry are revealing interesting new complexities and challenges, for example, the use of humanized mice, which have a metabolic phenotype that is neither fully human nor mouse [23]. New discoveries are revealing the consequences of virus-mediated metabolic rewiring on the process of viral pathogenesis, including susceptibility to infection, inflammation, disease progression, antiviral mechanisms, and controlling cell death processes. Considering the vast diversity of virus types and the wide range of cell types they infect, there will surely be many new discoveries of how the inert virus briefly comes “alive” through the metabolic manipulation of cells during infection.
